# Supplementary material for: HIVprotI: an integrated web based platform for prediction and design of HIV proteins inhibitors
Source: J Cheminform. 2018 Mar 9;10:12. doi: 10.1186/s13321-018-0266-y (PMC5845081; doi:10.1186/s13321-018-0266-y)
Supplement: Supplementary file 2 — Additional file 2. Source code of HIVProtI web server. [file 13321_2018_266_MOESM2_ESM.zip › HIVprotI_Source-code/hivprotI/weka-3-6-6/doc/weka/estimators/CheckEstimator.html]

CheckEstimator


---


|  |  |  |
| --- | --- | --- |
| **Overview** \| **Package** \| **Class** \| **Tree** \| **Deprecated** \| **Index** \| **Help** \| **Weka's home** | |  |
| PREV CLASS   **NEXT CLASS** | **FRAMES**    **NO FRAMES**     **All Classes** |  |
| SUMMARY: NESTED \| FIELD \| CONSTR \| METHOD | DETAIL: FIELD \| CONSTR \| METHOD |  |


---


## weka.estimators Class CheckEstimator

```
java.lang.Object
  weka.estimators.CheckEstimator
```

**All Implemented Interfaces:**: OptionHandler, RevisionHandler

---

``` public class CheckEstimator extends java.lang.Object implements OptionHandler, RevisionHandler ```

Class for examining the capabilities and finding problems with
estimators. If you implement a estimator using the WEKA.libraries,
you should run the checks on it to ensure robustness and correct
operation. Passing all the tests of this object does not mean
bugs in the estimator don't exist, but this will help find some
common ones.

Typical usage:

`java weka.estimators.CheckEstimator -W estimator_name
estimator_options` 

This class uses code from the CheckEstimatorClass
ATTENTION! Current estimators can only
1. split on a nominal class attribute
2. build estimators for nominal and numeric attributes
3. build estimators independendly of the class type
The functionality to test on other class and attribute types
is left in big parts in the code.
CheckEstimator reports on the following:

- Estimator abilities
  - Possible command line options to the estimator
  - Whether the estimator can predict nominal, numeric, string,
    date or relational class attributes. Warnings will be displayed if
    performance is worse than ZeroR
  - Whether the estimator can be trained incrementally
  - Whether the estimator can build estimates for numeric attributes
  - Whether the estimator can handle nominal attributes
  - Whether the estimator can handle string attributes
  - Whether the estimator can handle date attributes
  - Whether the estimator can handle relational attributes
  - Whether the estimator build estimates for multi-instance data
  - Whether the estimator can handle missing attribute values
  - Whether the estimator can handle missing class values
  - Whether a nominal estimator only handles 2 class problems
  - Whether the estimator can handle instance weights
- Correct functioning
  - Correct initialisation during addvalues (i.e. no result
    changes when addValues called repeatedly)
  - Whether incremental training produces the same results
    as during non-incremental training (which may or may not
    be OK)
  - Whether the estimator alters the data pased to it
    (number of instances, instance order, instance weights, etc)
- Degenerate cases
  - building estimator with zero training instances
  - all but one attribute attribute values missing
  - all attribute attribute values missing
  - all but one class values missing
  - all class values missing

Running CheckEstimator with the debug option set will output the
training and test datasets for any failed tests.

The `weka.estimators.AbstractEstimatorTest` uses this
class to test all the estimators. Any changes here, have to be
checked in that abstract test class, too.


Valid options are:

```
 -D
  Turn on debugging output.
```

```
 -S
  Silent mode - prints nothing to stdout.
```

```
 -N <num>
  The number of instances in the datasets (default 100).
```

```
 -W
  Full name of the estimator analysed.
  eg: weka.estimators.NormalEstimator
```

```
 Options specific to estimator weka.estimators.NormalEstimator:
```

```
 -D
  If set, estimator is run in debug mode and
  may output additional info to the console
```

Options after -- are passed to the designated estimator.

**Version:**
:   $Revision: 1.5 $

**Author:**
:   Len Trigg (trigg@cs.waikato.ac.nz), FracPete (fracpete at waikato dot ac dot nz)

**See Also:**: `TestInstances`

---

| **Nested Class Summary** | |
| --- | --- |
| `static class` | `CheckEstimator.AttrTypes`             class that contains info about the attribute types the estimator can estimate estimator work on one attribute only |
| `static class` | `CheckEstimator.EstTypes`             public class that contains info about the chosen attribute type estimator work on one attribute only |
| `class` | `CheckEstimator.PostProcessor`             a class for postprocessing the test-data |


**Constructor Summary**

`CheckEstimator()`


| **Method Summary** | |
| --- | --- |
| `void` | `doTests()`             Begin the tests, reporting results to System.out |
| `boolean` | `getDebug()`             Get whether debugging is turned on |
| `Estimator` | `getEstimator()`             Get the estimator used as the estimator |
| `static int` | `getMinMax(Instances inst, int attrIndex, double[] minMax)`             Find the minimum and the maximum of the attribute and return it in the last parameter.. |
| `int` | `getNumInstances()`             Gets the current number of instances to use for the datasets. |
| `java.lang.String[]` | `getOptions()`             Gets the current settings of the CheckEstimator. |
| `CheckEstimator.PostProcessor` | `getPostProcessor()`             returns the current PostProcessor, can be null |
| `java.lang.String` | `getRevision()`             Returns the revision string. |
| `boolean` | `getSilent()`             Get whether silent mode is turned on |
| `boolean` | `hasClasspathProblems()`             returns TRUE if the estimator returned a "not in classpath" Exception |
| `java.util.Enumeration` | `listOptions()`             Returns an enumeration describing the available options. |
| `static void` | `main(java.lang.String[] args)`             Test method for this class |
| `void` | `setDebug(boolean debug)`             Set debugging mode |
| `void` | `setEstimator(Estimator newEstimator)`             Set the estimator for boosting. |
| `void` | `setNumInstances(int value)`             Sets the number of instances to use in the datasets (some estimators might require more instances). |
| `void` | `setOptions(java.lang.String[] options)`             Parses a given list of options. |
| `void` | `setPostProcessor(CheckEstimator.PostProcessor value)`             sets the PostProcessor to use |
| `void` | `setSilent(boolean value)`             Set slient mode, i.e., no output at all to stdout |

**Methods inherited from class java.lang.Object**

`equals, getClass, hashCode, notify, notifyAll, toString, wait, wait, wait`

**Constructor Detail**

### CheckEstimator

```
public CheckEstimator()
```


**Method Detail**

### listOptions

```
public java.util.Enumeration listOptions()
```

:   Returns an enumeration describing the available options.

    :   **Specified by:**: `listOptions` in interface `OptionHandler`
    :   **Returns:**: an enumeration of all the available options.

---


### setOptions

```
public void setOptions(java.lang.String[] options)
                throws java.lang.Exception
```

:   Parses a given list of options.
    Valid options are:

    ```
     -D
      Turn on debugging output.
    ```

    ```
     -S
      Silent mode - prints nothing to stdout.
    ```

    ```
     -N <num>
      The number of instances in the datasets (default 100).
    ```

    ```
     -W
      Full name of the estimator analysed.
      eg: weka.estimators.NormalEstimator
    ```

    ```
     Options specific to estimator weka.estimators.NormalEstimator:
    ```

    ```
     -D
      If set, estimator is run in debug mode and
      may output additional info to the console
    ```

    :   **Specified by:**: `setOptions` in interface `OptionHandler`
    :   **Parameters:**: `options` - the list of options as an array of strings **Throws:**: `java.lang.Exception` - if an option is not supported

---


### getOptions

```
public java.lang.String[] getOptions()
```

:   Gets the current settings of the CheckEstimator.

    :   **Specified by:**: `getOptions` in interface `OptionHandler`
    :   **Returns:**: an array of strings suitable for passing to setOptions

---


### setPostProcessor

```
public void setPostProcessor(CheckEstimator.PostProcessor value)
```

:   sets the PostProcessor to use

    :   **Parameters:**: `value` - the new PostProcessor **See Also:**: `m_PostProcessor`

---


### getPostProcessor

```
public CheckEstimator.PostProcessor getPostProcessor()
```

:   returns the current PostProcessor, can be null

    :   **Returns:**: the current PostProcessor

---


### hasClasspathProblems

```
public boolean hasClasspathProblems()
```

:   returns TRUE if the estimator returned a "not in classpath" Exception

    :   **Returns:**: true if CLASSPATH problems occurred

---


### doTests

```
public void doTests()
```

:   Begin the tests, reporting results to System.out

---


### setDebug

```
public void setDebug(boolean debug)
```

:   Set debugging mode

    :   **Parameters:**: `debug` - true if debug output should be printed

---


### getDebug

```
public boolean getDebug()
```

:   Get whether debugging is turned on

    :   **Returns:**: true if debugging output is on

---


### setSilent

```
public void setSilent(boolean value)
```

:   Set slient mode, i.e., no output at all to stdout

    :   **Parameters:**: `value` - whether silent mode is active or not

---


### getSilent

```
public boolean getSilent()
```

:   Get whether silent mode is turned on

    :   **Returns:**: true if silent mode is on

---


### setNumInstances

```
public void setNumInstances(int value)
```

:   Sets the number of instances to use in the datasets (some estimators
    might require more instances).

    :   **Parameters:**: `value` - the number of instances to use

---


### getNumInstances

```
public int getNumInstances()
```

:   Gets the current number of instances to use for the datasets.

    :   **Returns:**: the number of instances

---


### setEstimator

```
public void setEstimator(Estimator newEstimator)
```

:   Set the estimator for boosting.

    :   **Parameters:**: `newEstimator` - the Estimator to use.

---


### getEstimator

```
public Estimator getEstimator()
```

:   Get the estimator used as the estimator

    :   **Returns:**: the estimator used as the estimator

---


### getMinMax

```
public static int getMinMax(Instances inst,
                            int attrIndex,
                            double[] minMax)
                     throws java.lang.Exception
```

:   Find the minimum and the maximum of the attribute and return it in
    the last parameter..

    :   **Parameters:**: `inst` - instances used to build the estimator: `attrIndex` - index of the attribute: `minMax` - the array to return minimum and maximum in **Returns:**: number of not missing values **Throws:**: `java.lang.Exception` - if parameter minMax wasn't initialized properly

---


### getRevision

```
public java.lang.String getRevision()
```

:   Returns the revision string.

    :   **Specified by:**: `getRevision` in interface `RevisionHandler`
    :   **Returns:**: the revision

---


### main

```
public static void main(java.lang.String[] args)
```

:   Test method for this class

    :   **Parameters:**: `args` - the commandline parameters


---


|  |  |  |
| --- | --- | --- |
| **Overview** \| **Package** \| **Class** \| **Tree** \| **Deprecated** \| **Index** \| **Help** \| **Weka's home** | |  |
| PREV CLASS   **NEXT CLASS** | **FRAMES**    **NO FRAMES**     **All Classes** |  |
| SUMMARY: NESTED \| FIELD \| CONSTR \| METHOD | DETAIL: FIELD \| CONSTR \| METHOD |  |


---
